# Heterogeneous (ITS-G5 and 5G) Vehicular Pilot Road Weather Service Platform in a Realistic Operational Environment

**DOI:** 10.3390/s21051676

**Published:** 2021-03-01

**Authors:** Muhammad Naeem Tahir, Marcos Katz

**Affiliations:** 1 Faculty of Information Technology and Electrical Engineering, University of Oulu, 90570 Oulu, Finland; 2 Center For Wireless Communication, University of Oulu, 90570 Oulu, Finland; marcos.katz@oulu.fi

**Keywords:** ITS, VN, V2V, V2I, 5GTN, ITS-G5, RWS

## Abstract

VANETs (Vehicular Ad hoc Networks) operating in conjunction with road-side infrastructure connecting road-side infrastructure are an emerging field of wireless communication technology in the vehicular communication’s domain. For VANETs, the IEEE 802.11p-based ITS-G5 is one of the key standards for communication globally. This research work integrates the ITS-G5 with a cellular-based 5G Test Network (5GTN). The resulting advanced heterogeneous Vehicular Network (VN) test-bed works as an effective platform for traffic safety between vehicles and road-side-infrastructure. This test-bed network provides a flexible framework to exploit vehicle-based weather data and road observation information, creating a service architecture for VANETs that supports real-time intelligent traffic services. The network studied in this paper aims to deliver improved road safety by providing real-time weather forecast, road friction information and road traffic related services. This article presents the implementation of a realistic test-bed in Northern Finland and the field measurement results of the heterogeneous VANETs considering the speed of vehicle, latency, good-put time and throughput. The field measurement results have been obtained in a state-of-the-art hybrid VANET system supporting special road weather services. Based on field measurement results, we suggest an efficient solution for a comprehensive hybrid vehicular networking infrastructure exploiting road weather information.

## 1. Introduction

Reliable communications are a fundamental requirement to ensure road traffic safety. Currently, road-side infrastructure, i.e., “Roadside Units (RSU) and Road Weather Stations (RWS), are one of the crucial parts of wireless sensor networks that can be utilized to observe and track vehicular activity along roads such as road conditions, speeding cars, car crashes and other dangerous situations. With the use of wireless sensor networks, the vehicles along the roads can have efficient and quick connection setups with additional internet gateways and fixed wireless access points (APs). The network increases road safety by providing the vehicle with different alerts such as traffic and weather information, as well as road condition information. For VANETs, IEEE launched the 802.11 standard for vehicular communications. The basic idea of this standard is to improve the safety applications for public as well as to improve the flow of road traffic in Vehicle-to-Vehicle (V2V) and Vehicle-to-Infrastructure (V2I) scenarios [1]. IEEE 802.11p is used as a base to the standardized ITS-G5, offering the GeoNetworking protocol for V2V and V2I communications. ITS-G5 is standardized by the European Telecommunications Standards Institute (ETSI). Cellular communication also plays an important role in Vehicular Networks (VNs) to exchange road weather and traffic information in order to enhance road traffic safety [2]. The 3rd Generation Partnership Project (3GPP) defined the V2X (Vehicle-to-Everything) communication specification based on LTE (Long Term Evolution) [3].

**Figure 1:**
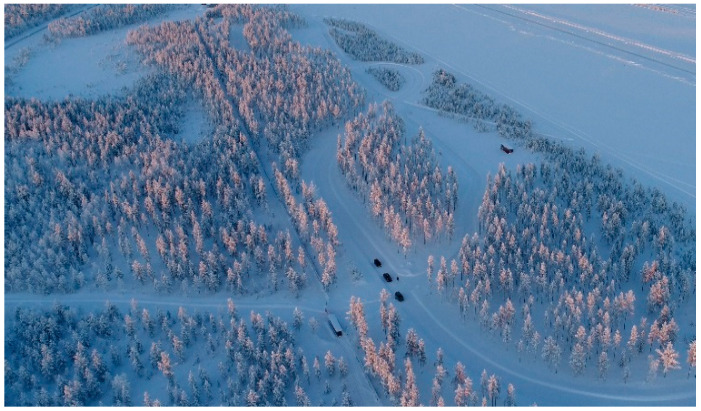
Heterogeneous vehicular networking test-track in Sodankylä, Finland

The Finnish Meteorological Institute (FMI) has developed a hybrid vehicular network infrastructure using ITS-G5 with a cellular-based 5G Test Network (5GTN). It provides an advanced, intelligent network containing heterogeneous networking capabilities for road traffic safety between vehicles and RSUs/RWSs. We used the sensor data of vehicles as well as the weather observation information from RWSs to develop a service architecture with the availability of real-time service capabilities. Mainly, we used commercial equipment, e.g., Sunnit briefcase and Cohda MK5 radio transceivers (Cohda Wireless, Wayville, Australia) to test the pilot system and to conduct field measurements in vehicular networking. The basic idea is to develop a state-of-the-art road traffic safety service architecture with accurate services such as location-based road weather data, forecast and accident alerts. This article also provides a platform for a real-time two-way communication with tailored pilot scenarios for vehicular networking. We conducted these pilot measurements using the Sod5G test-track in Sodankylä, Finland, as illustrated in Figure 1. The Sod5G test-track is majorly funded by the European Regional Development Fund (ERDF). The test-track has a length of 1.7 km and it is equipped with two road weather stations supporting an ITS-G5 protocol and a 5G test network base station together with different IoT sensors. This test-track offers the opportunity to design, develop, and test road weather services even in severe weather situations. In this article, we discuss the pilot measurements using ITS-G5 and 5GTN operability. The pilot scenarios and field measurements provide the base to plan, design, and develop the real-time intelligent road traffic system with a set of example services working in the heterogeneous ITS-G5 and 5GTN wireless technologies. The pilot system architecture and field measurement results are evaluated and compared with their expected results and requirements [4].

This paper is organized as follows. In Section 2, key properties of ITS-G5 and 5G standards are discussed, followed by Section 3, overviewing the ITS enabled services for VNs and the pilot system. In Section 4, the pilot system deployment and field measurement setup are considered, followed by Section 5, where pilot and field measurements are presented. Section 6 presents the results and analysis, followed by Section 7, where conclusions are drawn.

## 2. Vehicular Networking Wireless Technologies

### 2.1. ITS-G5

ITS-G5 is a European standard for vehicular communications based on the IEEE-1609.x and IEEE-802.11p standards. IEEE-802.11p operates at 5.850 GHz to 5.9250 GHz with data rate support between 3 and 27 Mbps in a 10 MHz channel bandwidth, and between 6 and 54 Mbps in a 20 MHz channel bandwidth. ITS-G5 supports a range of up to 1000 m in different environments such as rural, urban, suburban and highways supporting maximum relative vehicle speeds of 110 km/h [5]. The bandwidth of ITS-G5 can be selected according to the need of VANET requirements, either 10 MHz or 20 MHz channel bandwidth. The ITS-G5 standard also has a feature exploiting the Geo-Networking protocol for V2V and V2I communications. The ITS-G5 and Geo-Networking are standardized by the ETSI. ITS-G5 is based on the Media Access Control (MAC) and Physical (PHY) of IEEE 802.11p, as part of IEEE-802.11-2016. ITS-G5 defines the PHY and MAC layers of Open Systems Interconnection (OSI) architecture that relies on carrier sensing multiple access with collision avoidance (CSMA/CA) and orthogonal frequency division multiplexing (OFDM). ITS-G5 supports the asynchronous ad hoc protocol—a counterpart of the LTE-V2X synchronous ad hoc protocol with fixed, predefined time intervals [6].

### 2.2. 5G

The 5G is the fifth-generation cellular system that is based on mm-wave technology and will play a crucial role in vehicular communications; it is the latest cellular standard developed by the 3rd Generation Partnership Project (3GPP). The 5G standard is particularly designed to support high data rates (max. 20 Gbps) with a minimum latency for real-time application of 1 ms [7]. The 5G architecture also supports other evolving technologies, including mm-wave, Software-Defined Networks (SDNs), Device-to-Device (D2D) communications, Multiple-Input Multiple-Output (MIMO) systems, Network Function Virtualization (NFV), Heterogeneous Networks (HetNets), and network slicing. In [8], energy-efficient, software-defined vehicular edge networks are proposed to enable eco-routing and thus connected transportation systems. With the assistance of the above-mentioned technologies, 5G would be able to achieve very low end-to-end latency, high capacity, high data-rate, massive connectivity of devices, and reliable Quality of Experience (QoE) delivery.

Additionally, the network management is also a salient feature of 5G assisted by network slicing [9,10]. In [11], end-to-end network slicing is introduced to jointly optimize communication functionalities in both radio access and core networks, ensuring optimal data throughput and congestion-free systems. Due to the distinctive SDN’s capabilities of managing a large number of heterogeneous devices, operating in diverse network environments, and providing both improved security and flexible networking, 5G has a strong potential for VANETs communications [9].

## 3. ITS-Assisted Road Weather Services for VNs

**Figure 2:**
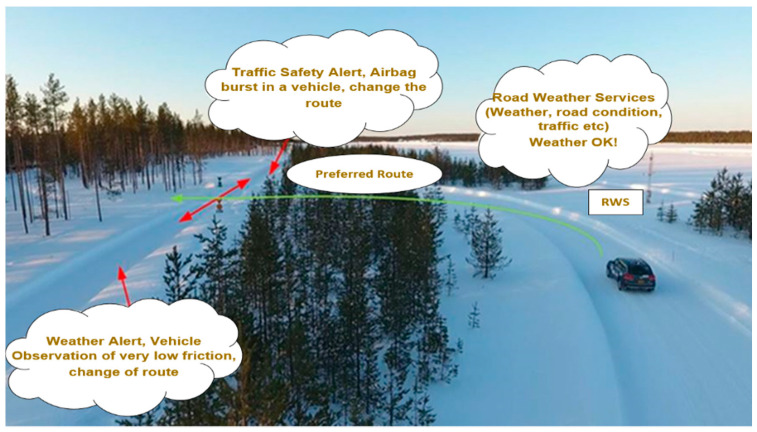
Road weather services tailored for pilot measurements of Intelligent Transport System (ITS)-G5 and 5G.

For pilot and field measurements, a test-track featuring an advanced state-of-the-art 5G test network and ITS-G5 was used. The network works as a testing platform to study, develop and pilot an advanced Intelligent Transport System (ITS) and real-time service architecture. The initial work on the 5G assisted road weather services was carried out in a previous initiative, namely the 5G Safe project, funded by Business Finland [5]. Providing an extra robustness for the information exchange between RWS and vehicles, 5G offers more refined road traffic climatic facilities. The test track infrastructure at the FMI supports different road weather services particularly designed to benefit VNs, as illustrated in Figure 2. Table 1 summarizes the considered road weather pilot services, including the real-time collection of weather information and alerts by using different IoT sensors. State-of-the-art equipment was used, including road friction instruments like Teconer RCM 411 (Teconer Oy, Helsinki, Finland) and WCM 411 (Teconer Oy, Helsinki, Finland) installed in vehicles and road weather station sensors including the Vaisala PWD-22, Vaisala DSC-111 (Vaisala Oyj, Vantaa, Finland), 2D Ultrasonic Anemometer, DST-111, 2*PT100, DRS-511, HMP45D, and a Zavio B7210 Full HD camera installed on the test-track. Ultimately, this real-time data is distributed to the nearby vehicles from RWSs for road traffic safety. Furthermore, the V2V and V2I communication in the 5G test network and the ITS-G5 were tested with a special “see-through” application, tailored to deliver vehicle camera data from the front of a vehicle queue during poor visibility conditions, making it possible to take precautionary measures for unexpected traffic anomalies. However, the pilot services were conducted using 5G and ITS-G5 on the test-track, which were performed and verified during the field measurements on the test-track at a conceptual level. In the near future, the pilot measurements will be further extended [10].

The vehicles receive the road traffic and weather services and gather observational information directly from the RWS and other vehicles. This information needs a high level of security and encryption. We also considered the security aspect of data. Indeed, data security was ensured before data broadcast and secure information management practices in the RWS, vehicles, and data clouds were implemented. We conducted an analysis to ensure that the generation of different weather service procedures were not corrupted or transferred without authorization. For data security, we are currently developing and contributing to different projects, offering security practices and methodologies for the VANETs use cases. One of the European union Electronic Components and Systems for European Leadership (EU-ECSEL JU) SafeCOP project at FMI designed and developed an extra safety layer for VANETs with an explicit run-time manager providing the validity and security of an individual communication object [11].

## 4. Pilot System and Field Measurements Setup

The pilot system and field measurements were carried out with ITS-G5 and 5GTN networking capabilities, and the test measurements were conducted on the aforementioned test-track. The field measurements of the pilot system evaluate the capability of vehicular networking considering V2V and V2I scenarios supporting special road weather services. The pilot system for field measurements is equipped with the Road Weather Stations (RWS) and the 5th generation Cohda MK5 On-Board Units (OBU) that is compatible with the ITS-G5 and Cohda MK6 OBU compatible to V2X applications providing the vehicle tracking, road traffic safety and efficiency. Both units have basically the same functionalities consisting of a Windows 7 workstation and Cohda Wireless MK5 transceivers tuned between 5.35 and 5.925 GHz, compatible with ITS-G5 (IEEE 802.11p) and V2X CTX-0800 OBU. The Cohda MK6 OBU is compatible with 5G and LTE bands together with the Global Navigation Satellite System (GNSS) feature. The configuration of the Cohda Wireless MK5 transceiver operates at a channel bandwidth of 20 MHz (10 MHz also available), designed on the 5.875–5.905 GHz band compatible with the ITS-G5 (IEEE 802.11p) and V2X OBUs. We used a SUNIT F-series vehicle PC (Sunit Oy, Kajani, Finland) for the user interface (UI) in vehicles during the pilot measurements. The PC integrates different modules between the telematics, windows, and display unit. It also provides a vehicle monitoring platform which enables the external vehicle sensors control. The configuration of the wireless transceiver units is as follows: operating band: 5.875–5.905 GHz and channel bandwidth of 20 MHz (10 MHz also available). The maximum data-rate of this channel operates is in the range 6–54 Mbps [12]. We expected to achieve the maximum attainable data rate, but the payload for the data packet (no framework) was restricted to 10 Mbps. The employed CTX-0800 OBU is compatible with 5G and LTE bands together with the GNSS feature. We used Iperf2 to transmit the UDP packets to the network, with a packet size of 1202 bytes and delay between packets set to 1 ms. The size of a data packet can be different, according to the requirements, and the specific packet size shows the standardized packet size of a road weather station. We used the standard transmit power of 21 dBm (in Europe) for packet transmission [13]. To receive and capture the UDP packets, we used the network protocol analyzer Wireshark. The analyzer gathers all the road weather and traffic information i.e., data packet capture time, average data-rate, average packet per second, average packet size, etc.). The analyzer also offers IO graphs, using which we can assess the captured data packet information as a time function. We used the network tool “Wireshark 3.4.3” to analyze the data packets on the network and transport layer of ITS reference layer model. For these field measurements, the test-track had no major traffic density or obstacles during the pilot system tailored along the test-track [13,14].

**Figure 3:**
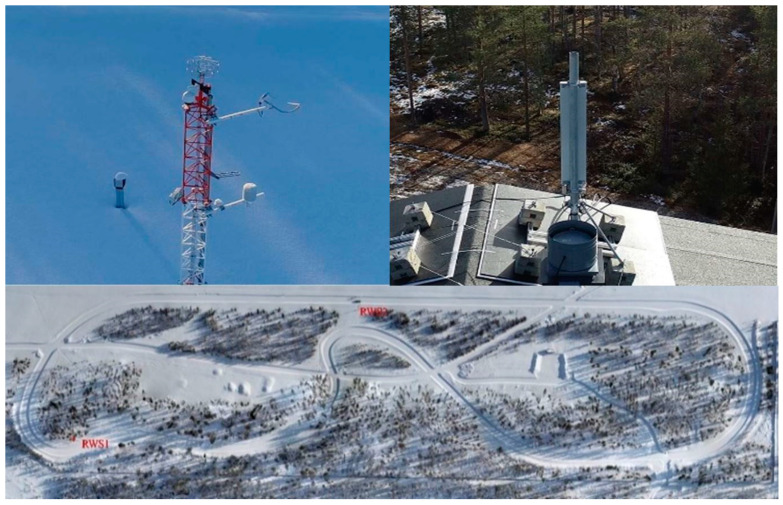
Test-track equipped with the 5G Test Network (5GTN) base station and Road Weather Stations (RWSs).

We tailored the field measurements in three different setups to analyze the performance of the heterogeneous VN in a realistic operational environment. These three different field measurement setups provide deep insight into network behavior at different vehicle speeds. In the V2I scenarios, we used a vehicle equipped with the OBU passing an RWS at speeds of 30, 40, and 50 km/h. Figure 3 shows the test-track equipped with the RWSs and a 5G base station for the field tests [6]. The RWSs transmit the data packets to the vehicle and we used a data packet capture software to capture ana analyze the packets. In the V2V scenario, we drove two OBU-equipped vehicles in opposite directions at speeds of 30, 40, and 50 km/h (single vehicle).

One of the vehicles transmitted data and the other vehicle received and captured the data. The first vehicle transmitted the data using only the OBU transmitter as a connection point with the second vehicle. In both scenarios, the transmitted UDP packet had a size of 1202 bytes with a delay of 1 ms, leading to almost a 10 Mbps data rate (max.). By using the ITS-G5 and 5GTN standards, the system’s theoretical range is from 50 to 1000 m, and the 5GTN range can be up to 1700 m (max.). In the V2I scenario, the vehicle connects to the RWS with a connection time *t_x_* given by

(1) $$t_{x\;}=\frac{2*r_{x\;}}{v_{x\;}},$$

where *v_x_* and *r_x_* represent the vehicle speed and RWS range, respectively. During the connection time *t_x_*, the throughput *T(tot)_x_* can be calculated as

(2) $$T_{x\;}=\frac{T{\left(tot\right)}_{x\;}}{t_{x\;}},$$

where *T(tot)_x_* is the throughput during the connection time *tx* and *Tx* is the time period through which packets were transmitted.

*T(tot)x = Px/tx* = total number of transmitted bits *(=no. of packets x length of packet)/tem-poral length duration of the transmission*.

For each speed, we have taken average across a number of measurements for average throughput.

Likewise, the V2V scenario used two vehicles and the connection time between two vehicles can be calculated while they are in range as

(3) $$t_{y\;}=\frac{r_{y\;}}{v_{y\;}},$$

where *v_y_* is the relative velocity of a vehicle and *r_y_* represents the range. The throughput *T(tot)_y_* between two vehicles at connection time *t_y_* is:

(4) $$T_{y\;}=\frac{T{\left(tot\right)}_{y\;}}{t_{y\;}},$$

where *T(tot)_y_* is the throughput during the connection time *t_y_* between vehicles and *T_y_* is the time period through which packets were transmitted.

*T(tot)_y_ = P_y_/t_y_* = total number of transmitted bits (=*no. of packets x length of packet)/temporal duration of the transmission*.

For each speed, we have taken an average across a number of measurements for average throughput.

The field measurements were tailored to calculate the pilot system capacity considering the average throughput. We also calculated the actual range of the VANET communication equipment, which was compared with the theoretical range [6,7].

For the V2I scenario, we conducted 25 measurement drives, and for the V2V scenario we conducted 30 measurements in two successive measurement sessions. The measurement conditions were the same for all field measurements. For the V2I and V2V scenarios, the vehicle passed the RWSs and the other vehicle by making a connection with RWSs and sustaining the connection within communication range with the maximum possible data rate [14,15,16,17,18,19,20].

## 5. Pilot Field Measurements

**Figure 4:**
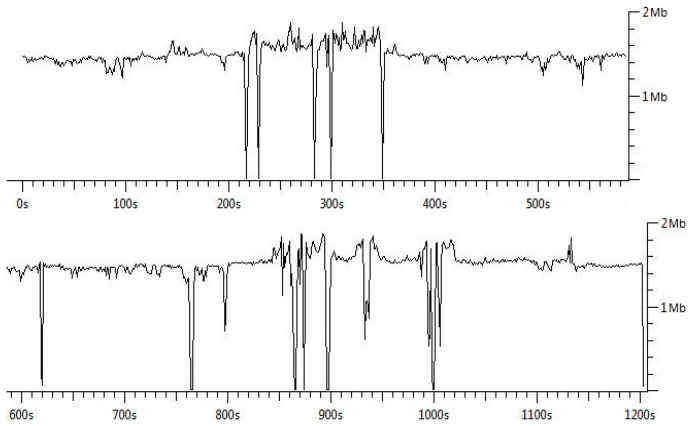
Data rate of vehicular networking using ITS-G5.

We started the pilot and field measurements of vehicular networking using the ITS-G5 network. While driving to the measurement area, we turned on the measuring devices, recorded the route and captured the packets. We conducted the field test measurements at three different speeds, namely 30, 40 and 50 km/h with an average speed of 40 km/h. The distance between cars was from 5 m to 200 m approximately. As shown in Figure 4, the connection between vehicles was on almost all the time and data rate stayed quite stable. The 1202 bytes of data packets were received with a 1ms transmission delay. The average throughput during the field measurement was 1.36 Mbps.

**Figure 5:**
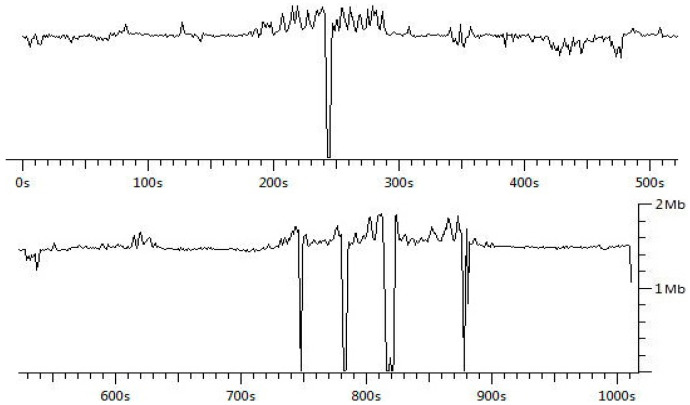
Data rate of vehicular networking using 5GTN.

In the second scenario, we conducted the field measurements of vehicular networking using the 5G test network. During the test measurements, the speed was from 0 to 50 km/h (average speed 40 km/h) and the distance between cars was approximately from 5 m to 200 m. As depicted in Figure 5, the connection between vehicles was on almost all the time and the data rate remained quite stable, as in the previous case. The 1202 bytes of data packets were transmitted with a 1 ms transmission delay. The average throughput during the trip was 1.55 Mbps.

**Figure 6:**
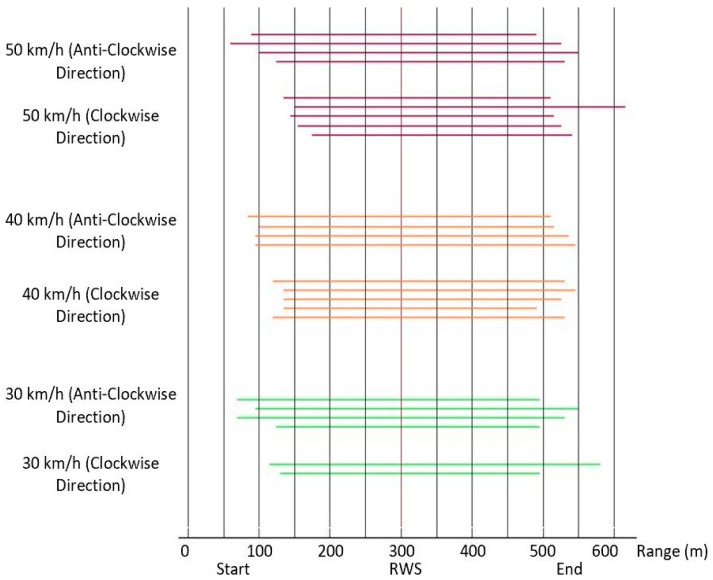
RWS communication range (m) on a test-track in Vehicle-to-Infrastructure communication (V2I) using heterogeneous networking.

To calculate the range of heterogeneous networking, we used a Python software where the RWS transmits the data to the nearby passing vehicle, as illustrated in Table 1. To calculate the range, we used Equation (5) by considering that the RWSs distributed the up-to-date road weather data to the nearby passing vehicles. The differences between ranges, calculated from the Wireshark measurements, were ±42 m. Figure 6 shows that the connections were established between 220 m and 480 m before the RWS and lost between 380 m and 630 m after the RWS. The lengths of the connections were from 710 m to 930 m for the considered 5GTN and ITS-G5 networks. The ideal situation for the communication link availability time was calculated according to (5), although the availability of a heterogeneous network has a relation between the ideal situation and the real-time field measurements [15,16].

The maximum range of the RWS (m) estimate was calculated as:

(5) $$Range=\frac{speed*\frac{\frac{measured\;Test\;drive\;length\;}{60}}{60}}{2},$$

where *speed* is given in km/h and *measured test drive length* is given in s.

**Figure 7:**
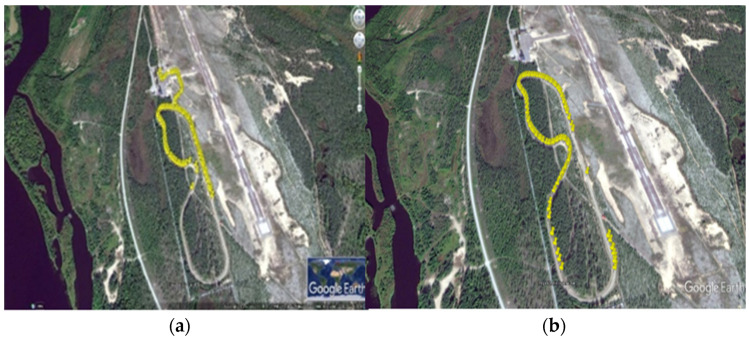
(**a**) RWS-1 packet capture using ITS-G5 (**b**) RWS-2 packet capture using ITS-G5.

In Figure 7a, we can see the UDP packet capture from RWS-1 and in Figure 7b we can see the UDP packet capture RWS-2, respectively. Both RWSs were implemented with ITS-G5 technology. The yellow spots represent the UDP packet capture during field measurements tailored on the test-track. Furthermore, vehicles encountering each other exchanged their latest road weather information received from the RWSs. Figure 7 reveals that there were some points where the connection was lost in V2V and V2I scenarios, and this ultimately affected the average throughput.

**Figure 8:**
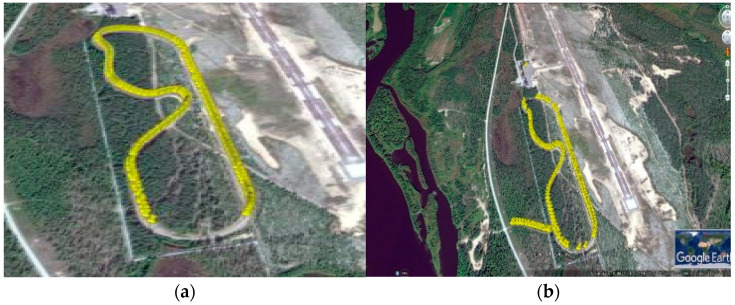
(**a**) Packet capture using 5GTN (**b**) Packet capture using heterogeneous (5GTN and ITS-G5) network.

In Figure 8a, the range of 5GTN packet capture during pilot measurements is shown together for the heterogeneous (ITS-G5 and 5GTN) network. Figure 8a shows that the 5GTN supported a greater range than ITS-G5 on the test-track for vehicular networking. The UDP packet capture in heterogeneous networking in Figure 8b illustrates that the network coverage and performance was enhanced in the field measurements. The yellow spots represent the UDP packet capture during field measurements tailored on the test-track. The missing yellow spots indicate the positions where data packets were lost.

## 6. Results and Analysis

In this section, we analyze the overall performance of the heterogeneous vehicular network in terms of average throughput, latency, packet size, and good-put time.

Table 2 illustrates the pilot measurement results of the V2I scenario, including the communication range in terms of VANET connection accessibility. The ideal situation for the communication link availability time was calculated according to (1), although the availability of a VANET communication link has a relation between the ideal situation and the collected results, respectively.

Table 3 summarizes the pilot measurement results in the tailored V2V scenario. The table provides the communication range as a VANET communication link availability using heterogeneous networking, calculated as a relation between the collected results and ideal situation based on (3).

For the V2I scenario, we conducted 25 measurement drives, and for the V2V scenario we conducted 30 measurement drives using the ITS-G5 and the 5GTN, respectively. In the heterogeneous network, the RWSs continuously transmit real-time road weather and traffic information, as presented in Table 1, and we captured the data packets in the testing vehicle for both V2V and V2I scenarios. For V2V and V2I scenarios, the testing vehicle was driving along the test-track. 

We evaluated the behavior of vehicular networking on the test-track, as presented in Table 2 and Table 3. We also analyzed the latency of the VANET communication on the test-track for V2I and V2V scenarios. Table 2 and Table 3 illustrate that the good-put time and latency affected the average throughput in heterogeneous vehicular networking. These results reveal that the network latency had an impact on the network throughput, while the V2V performance was slightly better as compared to the V2I scenario.

**Figure 9:**
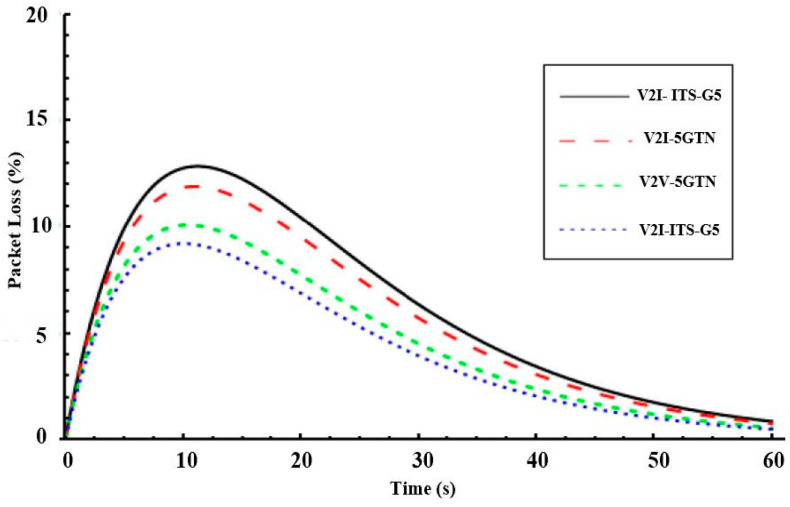
Packet loss between V2V and V2I using heterogeneous a Vehicular Network (VN).

Figure 9 shows the packet loss in vehicular networking during the pilot measurements. In the V2I scenario, the cellular system had a slightly higher packet loss compared to the ITS-G5 case, due to relatively long initialization time for the connection step-up in the field measurements. However, when connection between vehicles was established, the cellular network packet loss dropped dramatically, and it had almost the same performance at the end of the test drives. For the V2V scenario, the 5GTN performed better in contrast to the cellular system. Figure 9 also illustrates that the packet loss in the V2V scenario using ITS-G5 was slightly higher due to the haphazard nature of the test-track and weak communication link between vehicles due to the fluctuating distance between them. Features like high carrier frequency and edge computing made the 5GTN’s performance superior that of ITS-G5 (e.g., less latency). Hence, the overall performance of the pilot measurements illustrates that the heterogeneous network’s performance was good enough to fulfill the vehicular networking application requirements. This heterogeneous network shows that the pilot measurements performance have clear potential to decrease the accidents and loss of life on roads.

## 7. Conclusions

In this article, we illustrated and discussed the role of heterogeneous (ITS-G5/5GTN) vehicular communications in realistic operational environments as well as their pilot test results. We evaluated the capacity and range of a pilot system conducted with ITS-G5 and 5GTN in VNs. Based on capacity estimation, we effectively implemented a pilot system deployment assisted with an advanced road weather services in an operational environment at an FMI testing site. Based on the results from the pilot deployment and field tests, we proposed the deployment of a real-time ITS system architecture for VANET communications. The pilot system architecture offers a low-latency VANET networking experience to deliver real-time road weather services. With these facilities, FMI can test and analyze the ITS and road weather services. The field measurement results prove that the general behavior and performance of 5GTN was better than that of ITS-G5, and the performance was visibly better in the hybrid vehicular networking environment. The packet loss impacted the performance of the network and that ultimately affected the peak performance in terms of data throughput of 5GTN in the V2V scenario, but 5GTN performed better than the ITS-G5 network in the V2I scenario. The considered pilot system deployment proved that it can operate in real-time situations and that we can transmit the defined location-based pilot services accurately, aiming at decreasing road accidents.

## Figures and Tables

**Table 1:** Advanced ITS road traffic service.

ITS Services	RWS	Vehicle
Incident alert Blind-Spot-Collision-Warning (BCW)	Road surface condition sensors, humidity, wind, rain intensity and temperature	GPS and temperature
Incident alert Air-Traffic-Safety-Oversight (AOV)	-	V2V data through VANET networking
Road weather station (RWS) alerts	Road surface condition sensors, temperature, rain intensity, humidity, and wind	Highway, surface condition, temperature, GPS, and sensors
Itinerary weather	Road surface condition sensors, temperature, rain intensity, humidity, and wind	Highway surface condition, temperature, and GPS
Road Accident alert	-	Airbag burst, GPS, emergency lights on
Incident alert Slippery-road-Warning (SRW)	Road surface condition sensors, temperature, rain intensity, humidity, and wind	Road surface condition sensors, gyroscope, and GPS
Incident alert Road-Works-Warning (RWW)	Infrastructure to-vehicle information through Vehicular Ad hoc Network (VANET)	-

**Table 2:** Test measurement results for V2I.

Vehicle Speed (km/h)	Good-Put Time (s)	Data Packet Size (Bytes)	Latency (ms)	Avg. Throughput (Mbps)
30	28.95	1202	0.14	1.361
40	31.55	1202	0.23	1.225
50	26.21	1202	0.28	1.276

**Table 3:** Test measurement results for Vehicle-to-Vehicle communication (V2V).

Vehicle Speed (km/h)	Good-Put Time (s)	Data Packet Size (Bytes)	Latency (ms)	Avg. Throughput (Mbps)
30	42.437	1202	0.12	1.519
40	40.056	1202	0.20	1.361
50	32.561	1202	0.31	1.225

## Data Availability

Not applicable.
